# Higher blood volumes improve the sensitivity of direct PCR diagnosis of blood stream tuberculosis among HIV-positive patients: an observation study

**DOI:** 10.1186/s12879-015-0785-3

**Published:** 2015-02-06

**Authors:** Freddie Bwanga, Claudia Disqué, Michael G Lorenz, Vera Allerheiligen, William Worodria, Allan Luyombya, Irene Najjingo, Michael Weizenegger

**Affiliations:** Makerere University College of Health Sciences, Kampala, Uganda; MBN Clinical Laboratories, Kampala, Uganda; Molzym GmbH & Co. KG, Bremen, Germany; Hain Lifescience GmbH, Nehren, Germany; Medizinisches Versorgungszentrum Labor Dr. Limbach und Kollegen, Heidelberg, Germany

**Keywords:** Tuberculosis, Blood stream, Direct, Real time, PCR Diagnosis

## Abstract

**Background:**

Blood stream tuberculosis (TB), caused by *Mycobacterium tuberculosis* (MTB) is common among HIV-positive patients, turning rapidly fatal unless detected and treated promptly. Blood culture is currently the standard test for the detection of MTB in whole blood but results take weeks; patients deteriorate markedly and often die before a diagnosis of blood stream TB is made. Rapid molecular tests on whole blood, with potential for same day diagnosis of blood stream TB usually show low sensitivity due to the problem of insufficient MTB DNA template when extraction is performed directly on low blood volumes. This study assessed the influence of blood volume on the sensitivity of a HyBeacon PCR assay-the FluoroType® MTB (Hain Lifescience, Nehren, Germany) on direct detection of MTB in whole blood.

**Methods:**

Prospective recruitment of HIV-positive patients with clinical suspicion of blood stream TB but not on anti-TB or HIV drug treatment was done. Venous blood samples were collected and DNA extracted using the MolYsis (Molzym, Bremen, Germany) methods; for study A, from duplicate 1 ml (42 patients) and for study B (31 patients) from 9 ml EDTA blood samples. The FluoroType® MTB PCR assay targeting an IS6110 sequence was performed and results compared with blood culture.

**Results:**

The diagnostic sensitivity and specificity of the FluoroType® MTB PCR in study A was 33% and 97%, respectively. Corresponding values in study B were 71% and 96%, respectively. In both studies, one case each of blood culture-negative blood stream TB was detected with the FluoroType® MTB PCR assay. The median time to positivity of blood culture was 20.1 (range 12–32) for study A and 19.9 days (range 15–30) for study B.

**Conclusion:**

Larger blood volumes (9 ml) improved and gave acceptable sensitivity of direct PCR diagnosis of blood stream TB.

## Background

In the late 1980s, it was discovered that infection with the Human Immunodeficiency Virus (HIV) predisposed individuals to defects in the cell-mediated and humoral immunity resulting into susceptibility to blood stream infections with *Mycobacterium tuberculosis* (MTB), and to other pathogens such as non-typhoid salmonellae, *Streptococcus pneumoniae*, *Cryptococcus neoformans* and *Staphylococcus aureus* [1]. Since then, further studies have demonstrated the critical role of blood stream TB among the HIV patients [2-5]. Studies in Tanzania and Uganda reported MTB to be the most frequently isolated pathogen in 43% and 13% of positive blood cultures among the HIV-positive febrile patients, respectively [3,5]. However, blood cultures for *Mycobacterium tuberculosis* - a slow growing organism, requires around 3–6 weeks, a time period during which patients deteriorate markedly and may die before a diagnosis is made. Studies have reported the 30-day mortality attributed to blood stream TB to be at around 50% [5,6]. Thus, there is an urgent need for novel tests for rapid diagnosis of blood stream TB, particularly among the febrile HIV-positive patients.

In the past years, several rapid nucleic-acid amplification tests such as the Cepheid Xpert MTB/RIF and Line probe assays have been developed and approved by the WHO for the direct diagnosis of tuberculosis or drug resistant tuberculosis, particularly in sputum [7,8]. However, these assays are not adapted to whole blood and show poor diagnostic sensitivity for detection of MTB in whole blood [9].

New pre-analytic approaches aim at increasing the assay sensitivity through enrichment of microbial DNA from blood samples. Two commercial systems are available for this approach [10]. The LOOXTER (Analytic Jena, Jena, Germany) uses a protein that binds to methylated sites and thereby removes eukaryotic DNA providing enriched prokaryotic DNA to molecular testing. The other system, MolYsis (Molzym, Bremen, Germany) follows a protocol that involves lysis of blood cells, nucleolytic degradation of released human DNA and thereafter extraction and enrichment of microbial DNA. Here, we compared MolYsis-based extraction protocols for small (1 ml) and large blood volumes (9 ml) with respect to an effect on the detection sensitivity of mycobacteria by a HyBeacon PCR assay, FluoroType® MTB (Hain Lifescience, Nehren, Germany). For this purpose, blood from patients who were HIV sero-positive and who had clinical features of blood stream TB, as assessed by a senior infectious diseases physician was analyzed.

## Methods

### Study design

This was a cross-sectional study with prospective patient recruitment conducted in two sub studies (study A: February 2012-May 2012 and study B: November 2012-May 2013). The study was approved by the School of Medicine Research and Ethics Committee of Makerere University College of Health Sciences, approval number REC REF 2011-253.

### Study population

Participants in the studies were in-patients admitted at the medical wards of Mulago national referral and teaching Hospital Kampala, Uganda. The criteria for participant inclusion included HIV sero-positive, suspected on clinical assessment (on/off fever with night sweats for at least 2 consecutive weeks, loss of appetite associated with weight loss) to have blood stream TB infection, with or without features of pulmonary TB, and no history of TB or HIV drug treatment. Each study participant voluntarily signed a written consent to take part in the study before specimen collection.

### Specimen collection and processing

**Figure 1:**
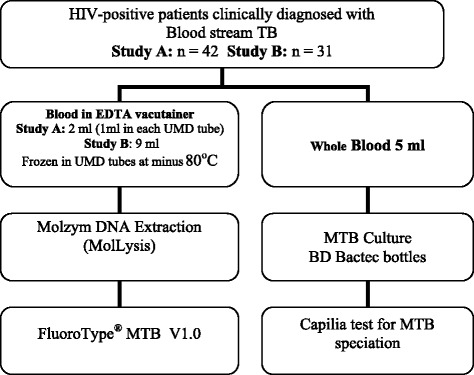
**Study summary chart.**

Specimens in this study included blood for direct mycobacteria PCR diagnosis and blood for MTB Blood culture as illustrated in Figure 1.

### Blood for direct mycobacteria PCR diagnosis

During studies A and B, 2 and 10 mls, respectively of whole blood were collected in EDTA vacutainers (BD, Heidelberg, Germany) and then transferred within two hours into UMD tubes (Molzym, Bremen, Germany). The contents were mixed with the cryoprotectant (0.4 ml) in the UMD tubes by vortexing thoroughly, and the tubes frozen at minus 80°C at the TB laboratory of the Department of Medical Microbiology Makerere University College of Health Sciences Kampala. These samples were later transferred to Bremen, Germany on dry ice for DNA extraction and molecular testing.

#### DNA extraction

Prior to testing of the clinical blood specimens, we studied the influence of blood volume on the detection of blood stream TB as follows: artificial spiking experiments of EDTA blood samples (1 ml and 10 ml) from a healthy individual were performed. For these experiments (conducted 3 times), 1 ml and 10 ml of whole blood was spiked with 30 and 3 cfu/ml of *Mycobacterium bovis* BCG, respectively. The blood specimens were inoculated with 10 μl of serial dilutions of a *Mycobacterium bovis* BCG culture and extracted following the protocols of the MTB-DNA Blood kit (Molzym).

For the clinical blood specimens, in study A, two 1 ml aliquots of a thawed UMD tubes were run through the small size sample DNA extraction protocol of the MTB-DNA Blood kit. In study B, the UMD tubes were thawed and the contents (9 ml) pipetted into a 50 ml centrifuge tube supplied with the MTB-DNA Blood kit. The protocol for large size samples was followed, and DNA was eluted with 0.1 ml of the supplied EB buffer.

#### Molecular analysis

DNA elutes from whole blood were analyzed using the FluoroType® MTB (FT MTB; Hain Lifescience). The assay consists of primers and a HyBeacon probe targeting the IS6110 element. The kit includes an internal amplification control. The assay was run on an optical thermo cycler (FluoroCycler®12, Hain Lifescience) with melt curve analysis for the specific detection of M. tuberculosis complex.

### Blood culture for MTB

Five millilitres of whole blood were aseptically drawn at venipuncture into BACTEC Myco/F-Lytic bottles and incubated in the Bactec 9120 Automated Blood Culture Instrument (Becton Dickinson, Sparks, Md) at the TB laboratory-Department of Medical Microbiology Makerere University College of Health Sciences Kampala until positive or for 42 days for negative cultures. Identification of *M. tuberculosis* from positive reported cultures was done with the Capilia test (Tauns Co. Ltd., Kamishima, Japan).

## Results

### Study population

HIV-positive patients aged 18 years and above, with clinical symptoms and signs of tuberculosis (TB) were used to determine the diagnostic sensitivity for detection of blood stream TB by PCR using small and large blood volume DNA extracts compared to blood culture detection. Study A was conducted from February 2012 until May 2012 and included 42 patients. Study B was performed from November 2012 until May 2013 and included 31 patients.

### Influence of blood volume on the detection of blood stream TB

Following the *Mycobacterium bovis* BCG spiking experiments, the FT MTB test yielded a positive result in the spiked blood samples in 3 of 3 repeat experimental assays. Two of the three experiments were still positive at 15 and 1.5 cfu/ml BCG with 1 ml and 10 ml extracts.

#### Study A results

The presence of MTB DNA in extracts from duplicate 1 ml blood samples was tested by FT MTB assay. Blood stream TB was diagnosed by culture in 9/42 cases (21%). FT MTB blood testing resulted in 4/42 positive cases (9%), of which 3 corresponded to culture-positive results (patients A11, A12, A15; Table 1). One patient (A10) was culture-negative and FT MTB-positive (Table 1). The diagnostic sensitivity and specificity of the FluoroType® MTB PCR assay in study A was 33% and 97%, respectively.

**Table 1:** **Patients in study A, positive for** ***M. tuberculosis*** **infection (n = 10)** ^**a**^

**Patient**	**Blood**	
	**Culture**	**FT MTB** ^**b**^
A7	+	-
A8	+	-
A9	+	-
A10	-	+
A11	+	+
A12	+	+
A13	+	-
A14	+	-
A15	+	+
A16	+	-

^a^+, positive;–, negative.

^b^In the case of positive results, at least one of the duplicate 1 ml samples was positive.

#### Study B results

In this study, 9 ml samples were extracted. The rate of blood culture positivity lay at 7/31 cases (22%). FT MTB blood testing resulted in 6 positive cases (overall rate, 19%) of which 5 were also positive by culture (patients B5, B6, B7, B9, B12). One patient (B10) tested blood culture-negative and FT MTB-positive, while two samples B8 and B11 were culture positive and FT MTB negative (Table 2). The diagnostic sensitivity and specificity of the FluoroType® MTB PCR assay in study B was 71% and 96%, respectively.

**Table 2:** **Patients in study B, positive for** ***M. tuberculosis*** **(n=9)** ^**a**^

**Patient**	**Blood**	
	**Culture**	**FT MTB** ^**b**^
B5	+	+
B6	+	+
B7	+	+
B8	+	-
B9	+	+
B10	-	+
B11	+	-
B12	+	+

^a^+, positive;–, negative.

^b^Nine milliliters blood extracted.

### Diagnostic values

The diagnostic performance of the FT MTB in blood samples was compared to blood culture results (Table 3). The sensitivity increased from 33% with duplicate 1 ml samples (study A) to 71% with 9 ml blood analyzed (study B). The specificities were comparably high in both studies-A (97%) and B (96%). Comparing 9 ml (study B) with duplicate 1 ml blood (study A) studies, other parameters including positive (83 vs. 75%) and negative predictive values (92 vs. 84%) and concordance of positive and negative results (90 vs. 83%) tended to be higher with the large blood volume analysis.

**Table 3:** **Diagnostic performance (%) of FT MTB assay against MTB blood culture as reference standard in relation to blood volume extracted**

	**TP**	**FP**	**FN**	**TN**	**Sensitivity, % (95% CI)**	**Specificity, % (95% CI)**	**PPV, %**	**NPV, %**	**Concordance, %**
**Study A (n=42)**	3	1	6	32	33(8–70)	97(84–100)	75	84	83
**Study B (n=31)**	5	1	2	23	71(29–96)	96(79–100)	83	92	90

CI=Confidence Interval, FN=False Negative, FN=False Positive, NPV=Negative Predictive Value, PPV=Positive Predictive Value, TN=True Negative, TP=True Positive.

## Discussion

This study was designed to evaluate the influence of sample volume used for the extraction of DNA from blood on PCR detection of MTB using FluoroType® MTB test. For this, two studies on HIV-positive patients with clinical features of blood stream TB were conducted. Blood culture diagnosis indicated comparable rates of blood stream TB in studies A (24%; see Table 1) and B (23%; see Table 2), which agree with the rates determined elsewhere employing HIV-positive patients in Tanzania (18%; 15).

Blood contains numerous PCR inhibitors, among them human DNA. Generally, removal of human DNA, which is in great excess to microbial DNA, positively influences the sensitivity of PCR amplification and sequencing of broad-range [11] as well as amplification of specific target sites [12,13]. This accounts also to MTB DNA detection in blood. Restrepo et al. noticed a positive effect on the sensitivity of MTB detection after separation of bacterial and host cell-rich fractions before DNA isolation [14]. Here we followed the approach of human DNA removal and selective enrichment of bacteria from blood for DNA extraction by a commercial kit. By extracting a large sample volume (9 ml), the FT MTB limit of detection (≤3 cfu/ml) could be decreased ten-fold compared to the 1 ml extraction protocol (≤30 cfu/ml). The large volume protocol also resulted in a higher positivity rate of other bacteria determined by the SepsiTest 16S rRNA gene assay and sequencing (Assay Bac, Molzym) [15] [16] in both studies (data not shown). This additional finding clearly shows that for the purpose of enhanced detection sensitivities, extraction of a larger blood volume is necessary.

The higher detection sensitivity with large than small sample volume extracts translated to improved diagnostic values. While FT MTB testing with 2x 1 ml blood extracts (study A) resulted in a rather poor sensitivity (33%; Table 3), the 9 ml extraction protocol (study B) provided a considerably higher diagnostic sensitivity (71%). This diagnostic sensitivity compares well with studies employing other molecular systems used for the identification of pathogens in blood [15,17]. The reason for the improved diagnostic sensitivity cannot be attributed solely to the large sample volume protocol inasmuch as in another study Feasey et al. [9] found a sensitivity of only 21% with the Cepheid Xpert MTB/RIF testing employing 18 ml blood samples. Rather, when considering similar detection limits of Xpert MTB/RIF (≤10 cfu/ml; 9) and FT MTB (≤3 cfu/ml; 10 ml protocol), the improved diagnostic sensitivity observed here follows the argumentation of a combined effect of the enrichment of pathogen DNA and the use of a large volume of blood [12]. A reason why FT MTB testing provided false negative results with 2/7 blood culture positive patients (study B; Table 2) may be attributed to the fact that the load of MTB in blood (median, 3–8 cfu/ml; 19) is at the limit of detection of the assay.

While blood culture is the most sensitive method, diagnosis of blood stream TB needs culturing for several weeks which delays in exclusion of the infection and appropriate therapy of infected patients [4]. Here, the median time to positive blood cultures took approx. 3 weeks (study A, 20.1 days [range, 12–32]; study B, 19.9 days [range, 15–30]; not shown). In contrast, FT MTB direct testing for blood stream TB can be completed within only 4.5 hours (2 h extraction, 2.5 h PCR analysis). For the current study, the assays were performed in Germany, but real-time PCR analysers are available in many laboratories in Uganda, and they are being used for similar testing here. Thus, from the standpoint of time-to-result, FT MTB testing can provide results in the 4.5 timeframe even in Uganda and it is superior to culturing in terms of time to clinically useful results. Another benefit of the FT MTB assaying of blood is the detection of culture negative blood stream TB (one each in both studies; see Tables 1 and 2). However, molecular testing may add additional costs to the standard culture test which, as is generally accepted and is shown also in this study, is obligatory for blood stream TB diagnosis. Nonetheless, FT MTB testing for blood stream TB can aid culture by the timely initiation of antimycobacterial therapy in positive cases.

## Conclusion

Analysis of MTB DNA from larger blood volumes (9 ml) improved and gave acceptable sensitivity of direct PCR diagnosis of blood stream TB.
